# *Salmonella* Agona Outbreak from Contaminated Aniseed, Germany

**DOI:** 10.3201/eid1107.041022

**Published:** 2005-07

**Authors:** Judith Koch, Annette Schrauder, Katharina Alpers, Dirk Werber, Christina Frank, Rita Prager, Wolfgang Rabsch, Susanne Broll, Fabian Feil, Peter Roggentin, Jochen Bockemühl, Helmut Tschäpe, Andrea Ammon, Klaus Stark

**Affiliations:** * Robert Koch Institute, Berlin, Germany;; † Robert Koch Institute, Wernigerode, Germany;; ‡ Public Health Department, State of Lower Saxony, Hannover, Germany;; § Institute for Hygiene and the Environment, Hamburg, Germany

**Keywords:** Pulsed-Field Gel Electrophoresis, Salmonella Agona, aniseed, outbreak, infants, herbal tea, Germany, case-control study, food safety

## Abstract

A nationwide outbreak of *Salmonella* Agona caused by aniseed-containing herbal tea occurred from October 2002 through July 2003 among infants in Germany. Consumers should adhere strictly to brewing instructions, although in exceptional cases this precaution may not be protective, particularly when preparing tea for vulnerable age groups.

*Salmonella enterica* serotype Agona is rarely isolated from humans in Germany (1). In other countries, *S*. Agona outbreaks among humans have been traced back to contaminated animal feed (2), dried milk, a peanut-flavored snack (3), and a cereal product (4).

In February 2003, a cluster of *S*. Agona infections was observed among children (median age 13 years, age range 3–20) receiving parenteral nutrition in an institution for handicapped persons in Lower Saxony, Germany. Analysis of national surveillance data showed a strong increase in *S*. Agona case reports in January and February 2003 compared to the same periods in 2001 and 2002. The increase was almost entirely attributable to infants ≤13 months of age. An outbreak investigation was conducted to identify risk factors and the vehicle of infection among infants.

## The Study

**Figure 1:**
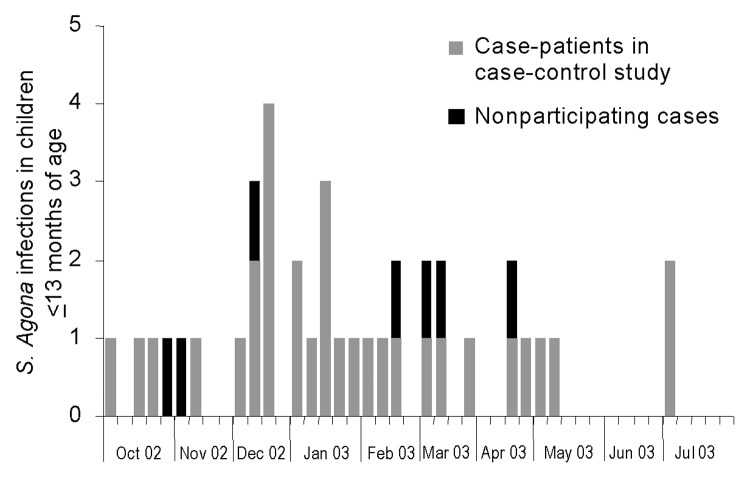
Epidemic curve showing week of onset of illness for confirmed cases of *Salmonella* Agona outbreak in infants ≤13 months of age, Germany, October 2002–July 2003 (n = 42).

From October 2002 through July 2003, a total of 42 *S*. Agona cases among infants ≤13 months of age were reported compared with 3 infections in this age group during the same period in the previous year (Figure 1). Cases occurred sporadically and were reported in 12 of the 16 German federal states. No substantial increase was found in the number of persons >13 months of age infected with *S*. Agona.

Among the 39 infants for whom data were available, 21 (54%) were girls, 23 (59%) were ≤6 months of age (median age 4.0 months), 35 (90%) had diarrhea, and 23 (59%) had fever. Twenty-one infants (54%) required hospitalization. No invasive disease was diagnosed and no deaths occurred. None of the patients had traveled abroad.

Exploratory interviews with the parents showed 2 common exposures: case-patients had received various brands of infant teas (n = 32, 82%) and had also consumed various milk powder products (n = 27, 69%) in the week before disease onset. Drinking teas containing herbs and spices such as fennel, aniseed, or caraway was reported for 27 (69%) infants. Because *S*. Agona had also been isolated from 2 aniseed samples in routine food safety monitoring in 2002 by the National Reference Center for Salmonella (Hamburg branch), a case-control study was launched to test the hypothesis that aniseed-containing herbal teas were the source of infection.

Patients ≤13 months of age with onset of diarrhea (defined as >2 soft stools in 24 hours) from October 1, 2002, to July 6, 2003, and *S*. Agona (outbreak strain) cultured from their stool were considered case-patients. Eight infants were excluded: 2 were from a set of triplets of whom only 1 was included, 3 had parents who could not be reached or refused to participate in the case-control study, 2 had an *S*. Agona pulsed-field gel electrophoresis (PFGE) patterns different from that of the outbreak strain, and 1 did not fulfill the case definition because of asymptomatic chronic infection. Controls were randomly selected from community population registries and frequency-matched by age group of the case-patients at time of illness (≤6 months or >6–≤13 months). The questionnaire elicited information on types of herbal teas, milk powder formulas, and other food consumed by the infants; tea preparation habits of parents; and breast-feeding history. For patients, details of usual food consumption were obtained for the 7-day period before disease onset. Parents of controls were asked about specific 7-day periods selected to match the distribution of the 7-day periods of the patients.

Overall, 31 patients and 130 controls were included in the study. Patients were significantly more likely than controls to have consumed any tea, any herbal tea from tea bags, and tea made from tea bags that contained aniseed (Table). Consumption of other types of tea bag products without aniseed or instant tea products was not associated with illness. Significantly fewer patients than controls were breast-fed. All other factors investigated were not significantly associated with illness.

**Table:** Univariate analysis of exposure factors for Salmonella Agona infection among infants ≤13 months of age, Germany, October 2002–July 2003

Exposure	Case-patients (n = 31)		Controls (n = 130)			
	No.	(%)	No.	(%)	Odds ratio	95% CI*
Any tea	30	(97)	67	(52)	28.2	3.7–213.1
Any herbal tea from tea bags	24	(77)	41	(32)	7.4	2.9–18.0
Tea from tea bags with aniseed	21	(68)	9	(7)	28.2	10.3–77.7
Tea from tea bags without aniseed	3	(10)	33	(25)	0.3	0.1–1.1
Instant tea	9	(29)	34	(26)	1.2	0.5–2.9
Breast feeding	6	(19)	67	(52)	0.2	0.1–0.7
	Subgroup cases† (n = 24)		Subgroup controls† (n = 41)			
Always used boiling water for tea preparation	16	(67)	35	(85)	0.3	0.1–1.2

*CI, confidence interval. †Subgroup analysis on case-patients and controls who drank tea made from tea bags.

Restricting analysis to those case-patients (n = 24) and controls (n = 41) who drank tea made from tea bags showed that the consumption of tea containing aniseed remained strongly associated with *S*. Agona infection (odds ratio [OR] 24.9, 95% confidence interval [CI] 6–102). Case-patients had consumed tea bag products containing aniseed from 12 different producers, and controls had consumed products from 8 different producers. Sixty-seven percent of the parents of patients reported always using boiling water for preparation of tea compared to 85% of control parents (p = 0.1).

When age in months was controlled in multivariable logistic regression, the consumption of tea from tea bags containing aniseed remained the only risk factor for *S*. Agona infection (OR 30.9, 95% CI 10.1–95.0). Breast-feeding was inversely associated with infection (OR 0.2, 95% CI 0.1–0.7).

The food safety authority in the state of Saxony-Anhalt collected 18 brands of teas containing aniseed from store shelves. One sample tested positive for *S*. Agona. In a subsequent nationwide sampling, various *Salmonella* serotypes were isolated from 61 (11%) of 575 tea and other aniseed-containing products. Tea from several of the contaminated tea brands had been drunk by affected infants. Among 44 *S*. Agona positive samples (8%), 41 were tea products containing aniseed and 3 were pure aniseed.

**Figure 2:**
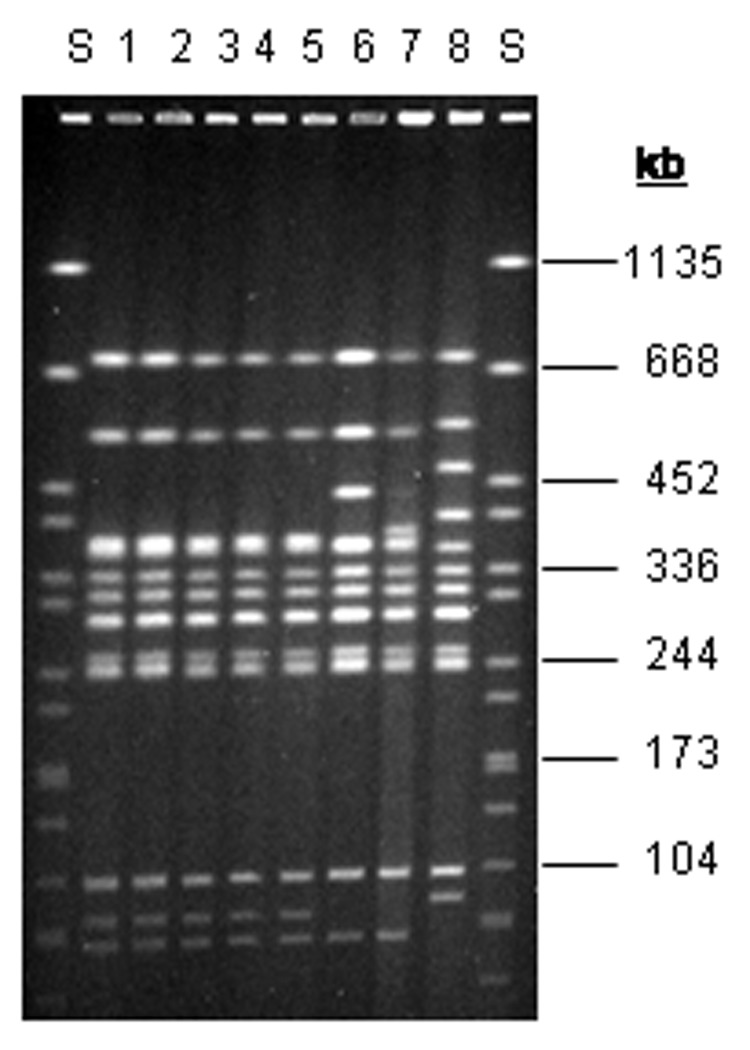
Pulsed-field gel electrophoresis patterns of *Xba*I-digested DNA from *Salmonella* Agona strains. Lanes 1 and 2, pattern SAX0001 (outbreak strain from tea); lanes 3–5, pattern SAX0001 (outbreak strain from humans); lanes 6–8 (nonoutbreak strains); lane S, molecular mass standard (*S*. Braenderup). kb, kilobases.

*S*. Agona isolates for subtyping were available from 17 patients, 6 different tea brands containing aniseed, and 3 samples of unprocessed aniseed. Molecular typing was performed at the National Reference Center for Salmonella (Wernigerode branch) by phage typing and PFGE. These methods are described elsewhere in detail (5). All isolates had phage type 02, identical PFGE patterns (Figure 2), and identical antimicrobial drug sensitivity patterns, but they were different from historical isolates. Four *S*. Agona–positive tea samples were quantitatively examined by using the most-probable-number method (6). This method yielded an estimated concentration of 0.036 salmonellae per gram of sample.

Products from all 12 producers of aniseed-containing herbal teas implicated in the study were traced back to a single importing company that had received the implicated large lot (≈15 metric tons) from Turkey. The company declared that the source of contamination of the raw product resulted from a batch of aniseed cultivated in Turkey that had been fertilized with manure.

All producers of tea products contaminated with *S*. Agona were notified by the food safety authorities. Unsold portions of contaminated production lots were recalled. The public was informed about the possibility that herbal tea may not be free from microbial contamination. Health authorities stressed that boiling water should be used in preparing tea and that a high steeping temperature should be maintained for at least 5 min before cooling the tea.

## Conclusions

This investigation provides strong epidemiologic and microbiologic evidence that herbal tea containing aniseed caused this diffuse outbreak of *S*. Agona among infants. Tea consumption was the only factor associated with illness in the study. Strains of *S*. Agona isolated from patients, aniseed-containing herbal tea, and unprocessed aniseed imported from Turkey showed an identical PFGE pattern. To our knowledge, this description is the first of a *Salmonella* outbreak caused by herbal tea.

Parents interviewed in this study indicated that herbal tea was not perceived as a product at risk for contamination with enteric pathogens, particularly since hot water is typically used in tea preparation. In Germany, aniseed-containing herbal teas, often in combination with fennel, are formulated and marketed specifically for their supposed antiflatulence and antispasmodic effects in infants. This may be 1 explanation of why this age group was particularly affected.

Aside from consumption patterns, host susceptibility likely played a role in this outbreak. Infants are particularly vulnerable to enteric pathogens because of factors such as gastric hypochlorhydria and insufficient mucosal immunity (7). Breast-feeding is known to reduce the severity of gastrointestinal infections among infants (8,9), which may explain the inverse association between a history of breast-feeding and *S*. Agona infection in this study.

Results of quantitative microbiologic investigations suggested low-level contamination of aniseed-containing teas with *S*. Agona. In previous outbreaks, similar low concentrations of salmonellae in foods (e.g., chocolate, cheddar cheese, and paprika-powdered potato chips) were reported, suggesting a low infectious dose (10–13). In dried food products such as aniseed, salmonellae can adapt to the dry state and may become resistant to environmental stress (e.g., heat, lack of nutrients) (14).

Two thirds of the parents of case-patients reported the consistent use of boiling water. Some parents reported quickly cooling the tea (e.g., by adding cold water). However, even if fewer parents of case-patients had always used boiling water (e.g., inaccurate recall), the use of boiling water may not have been sufficient to kill all viable salmonellae. Factors such as the addition of sugar, storage temperature, and elapsed time would have influenced the amount of salmonellae at the time of tea consumption. Further microbiologic studies on the heat resistance of salmonellae and desiccated strains are needed to provide information on how tea products can be rendered microbiologically safe through appropriate heat treatment during production and preparation at home.

Diffuse outbreaks may only be detected by demonstration of an identical pathogenic clone or when rare serotypes such as *S*. Agona are involved (15). Because of their wide distribution and long shelf-life, the implicated tea products could be sampled and linked to the human infections.

Because of underdiagnosis, the *S*. Agona cases in this outbreak most likely represented only a fraction of all infections due to contaminated herbal tea. Importing and tea-producing companies need to develop procedures to ensure microbiologic safety of their products. Brewing instructions on packages of tea should inform the consumer about potential microbiologic risks and the importance of following brewing instructions, especially in view of vulnerable populations such as infants and persons with weakened immune systems.
